# Extra-pleural pneumonectomy versus no extra-pleural pneumonectomy for patients with malignant pleural mesothelioma: clinical outcomes of the Mesothelioma and Radical Surgery (MARS) randomised feasibility study

**DOI:** 10.1016/S1470-2045(11)70149-8

**Published:** 2011-08

**Authors:** Tom Treasure, Loic Lang-Lazdunski, David Waller, Judith M Bliss, Carol Tan, James Entwisle, Michael Snee, Mary O'Brien, Gill Thomas, Suresh Senan, Ken O'Byrne, Lucy S Kilburn, James Spicer, David Landau, John Edwards, Gill Coombes, Liz Darlison, Julian Peto, for the MARS trialists

**Affiliations:** a Department of Mathematics, Clinical Operational Research Unit, University College London, London, UK; b Thoracic Surgery, Guy's and St Thomas' NHS Foundation Trust, London, UK; c Oncology, Guy's and St Thomas' NHS Foundation Trust, London, UK; d Department of Thoracic Surgery, Glenfield Hospital, Leicester, UK; e Department of Nursing, Glenfield Hospital, Leicester, UK; f Clinical Trials and Statistics Unit, Section of Clinical Trials, Institute of Cancer Research, Sutton, UK; g Thoracic Surgery, St George's Hospital, London, UK; h Radiology, Wellington Hospital, Wellington, New Zealand; i St James Institute of Oncology, St James's University Hospital, Leeds, UK; j Medical Oncology, Royal Marsden NHS Foundation Trust, Sutton, London, UK; k Oncology, Leicester Royal Infirmary, Leicester, UK; l Department of Radiation Oncology, VU University Medical Centre, Amsterdam, Netherlands; m Oncology, St James's Hospital and Trinity College Dublin, Ireland; n Thoracic Surgery, Northern General Hospital, Sheffield, UK; o Department of Non-communicable Disease Epidemiology, London School of Hygiene and Tropical Medicine, London, UK

## Abstract

**Background:**

The effects of extra-pleural pneumonectomy (EPP) on survival and quality of life in patients with malignant pleural mesothelioma have, to our knowledge, not been assessed in a randomised trial. We aimed to assess the clinical outcomes of patients who were randomly assigned to EPP or no EPP in the context of trimodal therapy in the Mesothelioma and Radical Surgery (MARS) feasibility study.

**Methods:**

MARS was a multicentre randomised controlled trial in 12 UK hospitals. Patients aged 18 years or older who had pathologically confirmed mesothelioma and were deemed fit enough to undergo trimodal therapy were included. In a prerandomisation registration phase, all patients underwent induction platinum-based chemotherapy followed by clinical review. After further consent, patients were randomly assigned (1:1) to EPP followed by postoperative hemithorax irradiation or to no EPP. Randomisation was done centrally with computer-generated permuted blocks stratified by surgical centre. The main endpoints were feasibility of randomly assigning 50 patients in 1 year (results detailed in another report), proportion randomised who received treatment, proportion eligible (registered) who proceeded to randomisation, perioperative mortality, and quality of life. Patients and investigators were not masked to treatment allocation. This is the principal report of the MARS study; all patients have been recruited. Analyses were by intention to treat. This trial is registered, number ISRCTN95583524.

**Findings:**

Between Oct 1, 2005, and Nov 3, 2008, 112 patients were registered and 50 were subsequently randomly assigned: 24 to EPP and 26 to no EPP. The main reasons for not proceeding to randomisation were disease progression (33 patients), inoperability (five patients), and patient choice (19 patients). EPP was completed satisfactorily in 16 of 24 patients assigned to EPP; in five patients EPP was not started and in three patients it was abandoned. Two patients in the EPP group died within 30 days and a further patient died without leaving hospital. One patient in the no EPP group died perioperatively after receiving EPP off trial in a non-MARS centre. The hazard ratio [HR] for overall survival between the EPP and no EPP groups was 1·90 (95% CI 0·92–3·93; exact p=0·082), and after adjustment for sex, histological subtype, stage, and age at randomisation the HR was 2·75 (1·21–6·26; p=0·016). Median survival was 14·4 months (5·3–18·7) for the EPP group and 19·5 months (13·4 to time not yet reached) for the no EPP group. Of the 49 randomly assigned patients who consented to quality of life assessment (EPP n=23; no EPP n=26), 12 patients in the EPP group and 19 in the no EPP group completed the quality of life questionnaires. Although median quality of life scores were lower in the EPP group than the no EPP group, no significant differences between groups were reported in the quality of life analyses. There were ten serious adverse events reported in the EPP group and two in the no EPP group.

**Interpretation:**

In view of the high morbidity associated with EPP in this trial and in other non-randomised studies a larger study is not feasible. These data, although limited, suggest that radical surgery in the form of EPP within trimodal therapy offers no benefit and possibly harms patients.

**Funding:**

Cancer Research UK (CRUK/04/003), the June Hancock Mesothelioma Research Fund, and Guy's and St Thomas' NHS Foundation Trust.

## Introduction

At a time when deaths from malignant pleural mesothelioma were rising in the UK^1^, ^2^ and Europe,^3^ data from the UK Thoracic Surgical Register of the Society for Cardiothoracic Surgery in Great Britain and Ireland showed that few patients were being offered surgery for their disease. Encouraging results from large case series had been reported for extrapleural pneumonectomy (EPP),^4^, ^5^, ^6^, ^7^ in which the lung and ipsilateral parietal pleura, pericardium, and hemidiaphragm are resected. In some institutions, this procedure, within a multimodal treatment regimen, became the standard of care in the management of patients with resectable malignant pleural mesothelioma.

In 2004, we did a systematic review to assess the available evidence for effectiveness of EPP.^8^ Median survival ranged from 17 to 35 months in seven surgical follow-up studies reported from 1999 to 2004. The available data had been reported retrospectively on the basis of completed treatment and so measurement of the extent to which survival was influenced by EPP itself rather than the initial selection of treatment and subsequent progressive selection for continued treatment in patients with a favourable prognosis was not possible.

To establish the efficacy of EPP, we designed the Mesothelioma and Radical Surgery (MARS) trial.^9^ EPP, within the context of trimodal therapy, was to be compared with induction chemotherapy but no EPP. At the start of the trial, a power calculation, on the basis of the difference claimed for effectiveness of EPP^8^ and natural history data,^10^ suggested that 670 patients would be needed to identify any statistically significant difference between EPP and no EPP with overall survival as the primary outcome. Because of the anticipated difficulty in recruiting patients, an initial feasibility study was done with the objective of randomising 50 patients within 1 year to EPP or no EPP to assess patient acceptability and to gauge the potential recruitment rate that could be expected in a larger trial. Randomisation between groups was possible but took longer than would be feasible to recruit sufficient numbers to a definitive trial.^11^ Here we report the survival and quality of life outcomes of MARS 2 years after recruitment was completed.

## Methods

### Patients

**Figure 1:**
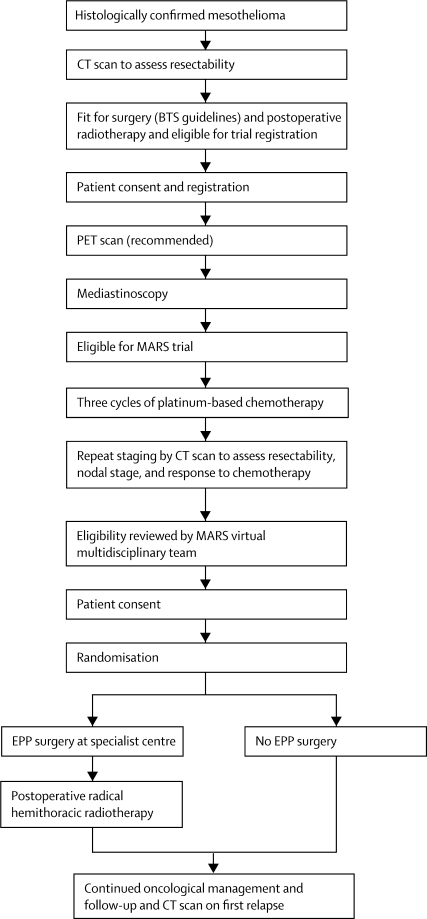
Trial design BTS=British Thoracic Society. MARS=Mesothelioma and Radical Surgery. EPP=extra-pleural pneumonectomy.

The MARS feasibility study was a multicentre randomised controlled trial with a prerandomisation registration phase and a two-stage consent process (figure 1). 12 UK hospitals took part in the study. Patients provided written informed consent before registration, after which they had surgical staging by cervical mediastinoscopy and, where available, PET. After staging, patients had three cycles of platinum-based chemotherapy with a regimen chosen by the treating physician at the local centre. Regimens suggested by the trial management group included mitomycin, vinblastine, and cisplatin; cisplatin and gemcitabine; or cisplatin and pemetrexed. Patients were informed that further written consent for randomisation between EPP or no EPP might be requested when they had completed chemotherapy and been reassessed clinically. Patients were eligible for registration if they were aged 18 years or older with pathologically confirmed mesothelioma and no evidence on preoperative CT staging of unresectable disease or distant metastases. They also had to be deemed fit enough to undergo preoperative chemotherapy followed by pneumonectomy (according to British Thoracic Society criteria for lung cancer surgery)^12^ and the planned postoperative radiotherapy.

After chemotherapy, patients underwent restaging by CT. Eligibility for EPP was then reassessed by the MARS virtual multidisciplinary team, which comprised a subset of the trial management group. This team was chaired by the chief investigator, coordinated by the trial team at the Institute of Cancer Research Clinical Trials and Statistics Unit (ICR-CTSU; Sutton, UK) and included the radiologist from the trial management group, the surgical coordinator, a medical and a radiation oncologist, and the designated MARS trial surgeon. For each patient deemed eligible by the referring clinical team, clinical data and imaging reports were circulated to MARS virtual multidisciplinary team members, and a teleconference was held to provide a consensus on whether that patient should be offered randomisation.

Study centres were also asked (but were not obliged) to complete a screening log of all patients with mesothelioma to document the eligible population and calculate the proportion who agreed to enter the registration phase of the study. Patients who fulfilled eligibility criteria and who received chemotherapy outside of the trial, but in a manner consistent with the protocol, could also be registered for assessment of eligibility for randomisation by the MARS virtual multidisciplinary team.

MARS was approved by Cambridgeshire 4 Research Ethics Committee (MREC/04/5/008) and was locally approved at all participating centres.

### Randomisation and masking

Patients were informed of the MARS virtual multidisciplinary team decision, and those still deemed eligible were invited to consent to be randomly assigned (1:1) to either EPP followed by radical radiotherapy or to no EPP. We used the TNM staging system proposed by the International Mesothelioma Interest Group.^13^ Patients were eligible for randomisation if they had completed preoperative chemotherapy and still had operable disease defined as T1–3, N0–1, M0.

Registration and randomisation were done by telephone to the ICR-CTSU. The randomisation sequence was generated by computer at the ICR-CTSU, with permuted blocks of varying size and stratification by surgical centre. No investigator or patient had access to the randomisation sequence. Patients and investigators were not masked to treatment allocation.

### Procedures

Patients assigned to EPP underwent surgery at one of the participating surgical centres in accordance with the trial surgical protocol (webappendix pp 1–2). Two surgical centres were designated initially because they had experienced surgeons who were used to undertaking EPP and whose results were audited. These centres remained the only surgical centres for much of the time MARS was open to recruitment. Three other centres were added later (Northern General Hospital, Sheffield, and St James's University Hospital, Leeds, opened to recruitment in 2006, but did not randomly assign an EPP patient until 2007), although in one there was no surgery within MARS. The surgeons at these centres also had experience of EPP, and their results had been audited. After surgery and providing the patient remained fit, postoperative radiotherapy was directed at the hemithorax (webappendix pp 3–5). All randomly assigned patients, including those in the no EPP group, received continued oncological management according to local policy, which could include chemotherapy, palliative radiotherapy, or further surgery.

We monitored acute and late radiotherapy effects (definded in the webappendix p 5), and collected clinical follow-up data including quality-of-life assessment by the European Organisation for Research and Treatment of Cancer (EORTC) QLQ-C30 and QLQ-LC13 questionnaires at registration, randomisation, 6 weeks, 3, 6, 9, 12, 18 (quality of life only), and 24 months, and annually thereafter.

The aim of the MARS feasibility study was to quantify the proportion of eligible patients subsequently randomised, to assess the feasibility of randomising 50 patients within 1 year,^11^ and to measure clinical outcomes in randomly assigned patients. Endpoints included the proportion randomised to EPP who completed trimodal therapy; perioperative mortality (defined for the purposes of referral to the independent monitoring committee for review as a death occurring during surgery, up to 30 days thereafter, or in a patient who was never discharged from hospital after the operation); quality of life; overall survival; progression-free or relapse-free survival, defined as time from randomisation to progression (no EPP patients) or relapse (EPP patients); or death from any cause. Deaths were reviewed by the independent data monitoring committee for relatedness to trial treatment.

### Statistical analysis

The main analyses of the MARS feasibility study were descriptive and included summary information from the screening logs on reasons for loss or withdrawal, the proportions of eligible patients registered and randomly assigned to treatment, and the proportion of randomly assigned patients who completed EPP surgery. We also assessed treatment compliance, complications, perioperative mortality and quality of life.

All analyses of randomly assigned patients were by intention to treat and were censored at the date last known to be alive. Survival rates were compared by the exact log-rank test because of the small patient numbers. HRs and 95% CIs were calculated by Cox proportional hazards regression, adjusting for the prespecified prognostic factors of sex, histological subtype, stage at randomisation, and age at randomisation. HRs less than 1·0 favoured EPP. We used the reverse Kaplan-Meier method to calculate median follow-up. All proportions are reported with two-sided 95% CIs. All analyses were done with Stata (version 10.1).

During the trial, safety and efficacy data were reviewed regularly by the independent data monitoring committee. Perioperative mortality was monitored by a group sequential approach with the potential for the independent data monitoring committee to advise on trial closure should a stopping boundary be crossed. This trial is registered, number ISRCTN95583524.

### Role of the funding source

The sponsor of the study had no role in study design, data collection, data analysis, data interpretation, or writing of the report. The corresponding author had full access to all the data in the study and had final responsibility for the decision to submit for publication.

## Results

**Figure 2:**
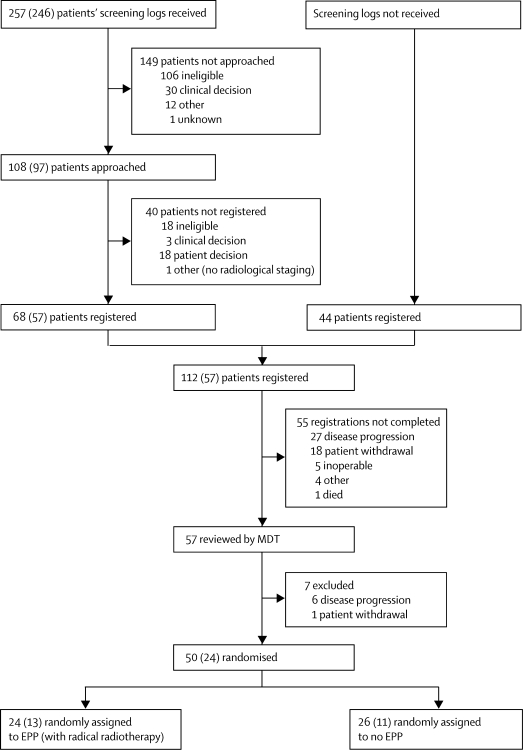
Feasibility of registration and recruitment to MARS Numbers in brackets indicate those patients screened who did not have chemotherapy before registration. MARS=Mesothelioma and Radical Surgery. MDT=multidisciplinary team. EPP=extra-pleural pneumonectomy.

Between Oct 1, 2005 and Nov 3, 2008, 112 patients were registered, of whom 50 were subsequently randomly assigned to EPP (n=24) or to no EPP (n=26; figure 2). For this analysis, all treatment data were included up to Nov 23, 2009; outcome data were included up to April 19, 2010. The median time between registration and randomisation was 3·6 months (IQR 2·8–4·3). Median follow-up from randomisation, in all patients, was 24·7 months (IQR 21·6–32·2). 62 patients (55·4%) did not proceed to random allocation, mainly because of disease progression (33 patients), inoperability (five patients), or patient choice (19 patients).

12 centres submitted screening logs for 257 patients. Figure 2 shows the numbers of patients registered and subsequently randomly assigned to treatment among those who were screened. Of the 246 who were screened before any chemotherapy, 97 (39·4%, 95% CI 33·1–45·7) were invited to enter the registration phase, 57 (23·2%, 18·0–28·8) were registered, and 24 (9·8%, 6·4–14·2) were subsequently randomised. 136 of 257 (53%) patients were excluded for clinical reasons (ineligibility, clinical decision, disease progression, and inoperability) before the patient was approached, 21 of 108 (19%) were excluded before registration, and 39 of 112 (35%) were excluded before randomisation. These data suggest an unavoidable cumulative loss of 76·3% (95% CI 70·6–81·3) independent of patient withdrawal or other reasons.

During the recruitment period an increasing number of patients were referred to MARS centres for assessment for eligibility. 44 patients were registered in this way, bypassing the screening log process, and therefore came from an unknown denominator.

Table 1 shows patient characteristics at registration for all 112 registered patients and the 50 who were subsequently randomised. 83 (74%) of 112 registered patients received three cycles of chemotherapy. Seven patients had more than three cycles, two of whom were deemed eligible by the MARS virtual multidisciplinary team and subsequently randomly assigned, and four patients had fewer than three cycles of chemotherapy, one of whom was subsequently randomly assigned. 18 patients (16%) did not receive any chemotherapy, most frequently because of disease progression, and were thus ineligible for random allocation. The most common chemotherapy regimen given was cisplatin and gemcitabine (38 of 94; 40%), followed by cisplatin and pemetrexed (24; 26%), and mitomycin, vinblastine, and cisplatin (20; 21%).

**Table 1:** Patient characteristics at registration

		**Overall cohort (n=112)**	**Patients subsequently randomised (n=50)**
Sex (male)		101 (90%)	46 (92%)
Age at registration (years)		61·7 (5·3)	61·5 (4·4)
WHO performance status score			
	0	34 (30%)	19 (38%)
	1	68 (61%)	28 (56%)
	Unknown	10 (9%)	3 (6%)
Method of histological diagnosis			
	Abram's needle or blind biopsy	2 (2%)	0 (0%)
	CT or ultrasound guided	27 (24%)	10 (20%)
	Surgical	76 (68%)	38 (76%)
	Other*	3 (3%)	2 (4%)
	Unknown	4 (4%)	0 (0%)
Histological subtype			
	Epithelioid	83 (74%)	40 (80%)
	Sarcomatoid	3 (3%)	0 (0%)
	Mixed or biphasic	12 (11%)	7 (14%)
	Unknown on first relapse	14 (13%)	3 (6%)

Data are number (%) or mean (SD). Percentages do not sum to 100 in some cases because of rounding.

* Aspiration cytology (n=1), cytology (tumour block; n=1), and pleural effusion (n=1).

38 (76%) of 50 patients subsequently randomly assigned to EPP (n=18) or no EPP (n=20) had PET-CT scan information available for the MARS virtual multidisciplinary team. PET-CT scan results suggested that 23 patients (ten EPP and 13 no EPP) were resectable, five (four EPP and one no EPP) were equivocal, and in ten patients (four EPP and six no EPP) this information was not available. Table 2 shows the characteristics of the 50 patients at random allocation. No differences in terms of stage or other patient-related features were noted between the two groups.

**Table 2:** Characteristics of patients subsequently randomly assigned to EPP or no EPP

		**EPP (n=24)**	**No EPP (n=26)**
Sex (male)		23 (96%)	23 (88%)
Age group at registration (years)			
	<45	0 (0%)	0 (0%)
	45–54	2 (8%)	2 (8%)
	55–64	17 (71%)	19 (73%)
	65–74	5 (21%)	5 (19%)
WHO performance status score at randomisation			
	0	13 (54%)	10 (38%)
	1	11 (46%)	15 (58%)
	Missing	0 (0%)	1 (4%)
Method of histological diagnosis			
	Abram's needle or blind biopsy	0 (0%)	0 (0%)
	CT or ultrasound guided	4 (17%)	6 (23%)
	Surgical	20 (83%)	18 (69%)
	Other*	0 (0%)	2 (8%)
	Unknown	0 (0%)	0 (0%)
Histological subtype			
	Epithelioid	20 (83%)	20 (77%)
	Sarcomatoid	0 (0%)	0 (0%)
	Mixed or biphasic	3 (13%)	4 (15%)
	Unknown	1 (4%)	2 (8%)
Stage* at randomisation			
	T1, N0, M0	3 (13%)	4 (15%)
	T2, N0, M0	12 (50%)	12 (46%)
	T2, N1, M0	0 (0%)	1 (4%)
	T3, N0, M0	9 (38%)	8 (31%)
	T3, N1, M0	0 (0%)	1 (4%)
Chemotherapy received†			
	Cisplatin and gemcitabine	10 (42%)	10 (38%)
	Cisplatin and pemetrexed	8 (33%)	8 (31%)
	Mitomycin, vinblastine, and cisplatin	6 (25%)	5 (19%)
	Cisplatin and vinorelbine	0 (0%)	3 (12%)
Change in tumour stage during chemotherapy			
	Responded	3 (13%)	2 (8%)
	Stable	15 (63%)	19 (73%)
	Progressed	3 (13%)	4 (15%)
	Missing	3 (13%)	1 (4%)

Data are number (%). Percentages do not sum to 100 in some cases because of rounding. EPP=extra-pleural pneumonectomy.

* Combination of the staging results by CT scan, PET scan, and mediastinoscopy.

† Clinicians were free to choose the chemotherapy regimen as long as it included a platinum-based drug.

**Figure 3:**
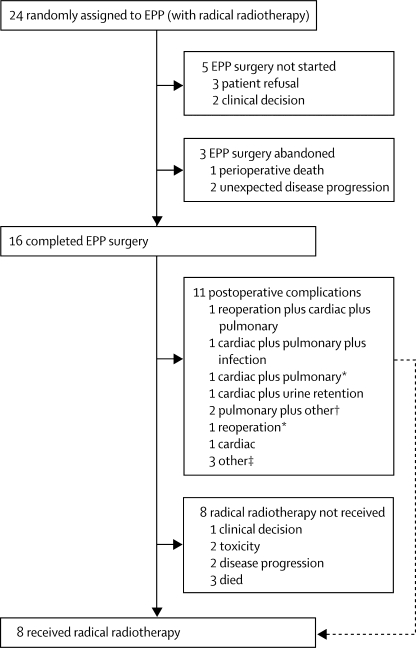
Feasibility of EPP surgery and radical radiotherapy treatment EPP=extra-pleural pneumonectomy. *Subsequent perioperative death. †Other complications were flexible bronchoscopy or drain infection in pneumonectomy cavity; ischaemic right leg requiring femoropopliteal bypass and eventual below knee amputation with culture-positive pneumonia needing mini tracheostomy. ‡Postoperative pain, low blood pressure, and intraoperative bleeding and further bleeding from chest drains postoperatively.

Of the 24 patients randomly assigned to EPP, five did not proceed to surgery: three by patient choice and two by clinician decision (figure 3). EPP was started in 19 patients but in two patients with unexpected disease progression EPP was not completed and one patient died during surgery. Thus, 16 patients completed EPP surgery (figure 3). Four surgeons did the EPP operations: two were assigned ten each before randomisation, one assigned three, and one assigned one. If the patient was not randomly assigned to receive surgery or declined, the surgeon did not do an EPP. There were two further deaths within the protocol-defined perioperative period, giving a total of three perioperative deaths in 24 (12·5%, 95% CI 2·7–32·4) in patients randomised to EPP by intention to treat and three perioperative deaths in 19 (15·8%, 3·4–39·6) patients in whom EPP was attempted. 11 of 16 patients who were assigned to and completed EPP surgery had at least one postoperative complication (figure 3).

Eight of the 16 patients who completed EPP received radical radiotherapy, five of whom had complications. Severe (grade 3 or 4) acute radical radiotherapy side-effects were rare: two patients had grade 3 fatigue and one had grade 3 pain. Severe late side-effects were fatigue (n=1, grade 3), pneumonitis or dyspnoea (n=2, grade 3), and ascites (n=1, grade 3). One patient developed paraplegia 42 days after completion of radiotherapy; this patient had MRI and clinical features of herpes myelitis (grade 4). This patient also had coexisting herpes retinitis and progressive diffuse changes on MRI of the spinal cord outside the irradiated region and thus the diagnosis of radiation-induced myelopathy was excluded. The patient was still paraplegic with no evidence of recurrence 2·3 years after multimodal treatment.

Six patients allocated to EPP received additional oncological management. Of these, two had completed EPP surgery and none received radical radiotherapy. One patient whose EPP operation was not started received further chemotherapy. One patient whose EPP operation was abandoned received radiotherapy. In two patients a decision to do lung-sparing debulking or pleurectomy surgery was taken during the operation. Two patients who had completed EPP operation had further surgery to deal with thoracic space infection.

16 of the 26 patients randomly assigned to no EPP received further oncological management: one received radiotherapy alone; seven had further chemotherapy alone; one had EPP surgery off trial; one had radiotherapy and chemotherapy; one had radiotherapy and non-EPP surgery; two had chemotherapy and EPP surgery off trial; one had chemotherapy and cediranib (as part of a phase 1 trial); and two had radiotherapy, chemotherapy, and non-EPP surgery. Thus, three patients had EPP off trial, 13 had further chemotherapy, five had some form of radiotherapy, three had non-EPP surgery, and ten received no further treatment.

At a median follow-up from randomisation of 24·7 months (IQR 21·6–32·2), 30 of 50 patients had died (EPP n=17; no EPP n=13); four of these deaths (three in the EPP group and one in the no EPP group) occurred more than 18 months after randomisation. 25 deaths were due to mesothelioma (EPP n=13; no EPP n=12), one (EPP) was due to respiratory failure before relapse, and four were perioperative (EPP n=3; no EPP n=1). Of the perioperative deaths in patients randomly assigned to EPP, one had a rupture of the aortic isthmus (multiple sites) and died on the operating table; one died at home (cause unknown) shortly after a further operation to have a diaphragm patch repaired; and one died of bronchopneumonia 6 weeks after the EPP operation. The perioperative death in the no EPP group was one of the patients who underwent EPP surgery outside the trial; the patient died of multiple organ failure.

**Figure 4:**
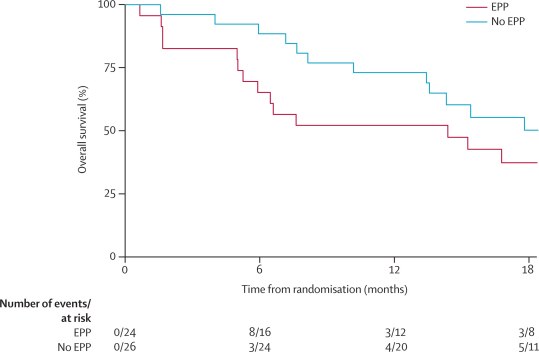
Overall survival EPP=extra-pleural pneumonectomy.

12-month survival was 52·2% (95% CI 30·5–70·0) in those allocated EPP and 73·1% (51·7–86·2) in those allocated to no EPP (difference 18·0%, −1·8 to 43·9; figure 4). The hazard ratio for overall survival in the EPP group (unadjusted) versus the no EPP group was 1·90 (95% CI 0·92–3·93; exact p=0·082). After adjustment for prespecified prognostic factors the HR was 2·75 (1·21–6·26; p=0·016). Median survival from randomisation for patients allocated to EPP was 14·4 months (5·3–18·7). For patients randomised to no EPP, median survival was estimated to be 19·5 months (13·4 to time not yet reached). None of the three long-term survivors allocated to no EPP crossed over to EPP.

**Figure 5:**
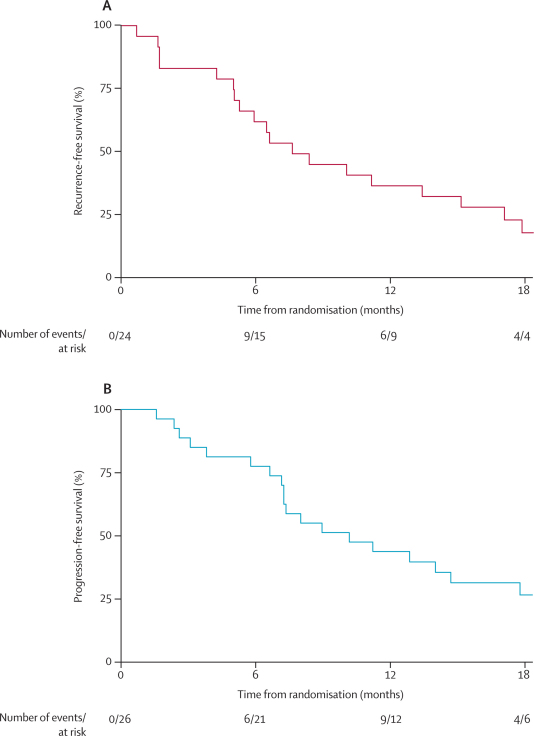
Recurrence-free and progression-free survival Recurrence-free survival in patients randomised to EPP (A) and progression-free survival in patients randomly assigned to no EPP (B). EPP=extra-pleural pneumonectomy.

42 of 50 patients (EPP n=19, no EPP n=23; four from the no EPP group had an event more than 18 months after random allocation) had disease recurrence (EPP group), progression (no EPP group), or died before relapse or progression (figure 5). 12-month recurrence-free survival in the EPP group was 34·8% (95% CI 16·6–53·7) and median recurrence-free survival was 7·6 months (5·0–13·4). 12-month progression-free survival in the no EPP group was 42·3% (23·5–60·0) and median progression-free survival was estimated to be 9·0 months (7·2–14·7).

**Figure 6:**
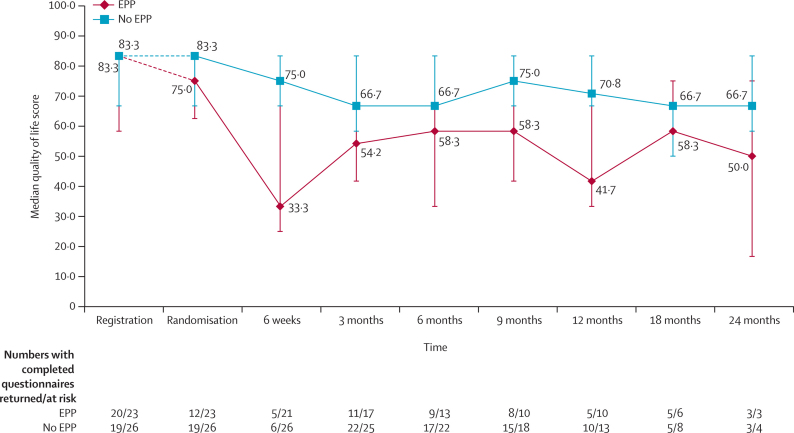
Quality of life

23 patients in the EPP group and 26 in the no EPP group consented to quality-of-life assessment and 12 and 19 patients completed the quality-of-life questionnaires, respectively. Median quality-of-life scores seemed to be lower for the EPP group than the no EPP group, with the lowest median score shortly after surgery (figure 6); however, there were no statistically significant differences between treatment groups.

12 serious adverse events were reported during the study period: ten in the EPP group and two in the no EPP group. Three were suspected unexpected serious adverse reactions (two in the EPP group and one in the no EPP group), eight were serious adverse reactions (all in the EPP group), and there was one other serious adverse event in the no EPP group.

## Discussion

In an intention-to-treat analysis of outcome data in the MARS trial, we noted no survival advantage for the EPP surgery group compared with the no EPP group. Furthermore, when adjusted for prognostic variables, patients allocated to no EPP had a better outcome than those allocated to EPP. No significant differences between groups were reported in the quality-of-life analyses. In summary, the outcomes of MARS provide no evidence of benefit from EPP within trimodal therapy over chemotherapy alone, for survival or quality of life.

Survival data for EPP were provided in a recent systematic review,^14^ which allows us to put the EPP survival data from MARS in context (panel). For 30 studies where data were given, 12-month survival ranged from 36% to 83%, with an overall survival of 57·1% (1231 of 2155 patients) compared with 52·2% in the MARS EPP arm. For 35 studies that included 2314 patients, median survival ranged from 4 to 35 months, with a median of 14·5 months. A subset of four of these studies^15^, ^16^, ^17^, ^18^ plus an EORTC phase 2 trial^19^ are most comparable with MARS in that patients were all operated on since 2000, had similar stage criteria for EPP, and were planned for trimodal therapy in the same sequence, starting with chemotherapy with subsequent EPP and then radiotherapy (table 3). Median survival by intention to treat ranged from 14 to 25·5 months. In studies in which chemotherapy was the first modality, the survival time was counted from the start of chemotherapy^15^, ^16^, ^17^, ^18^ or from registration.^19^ In MARS, survival was calculated from the later timepoint of randomisation to EPP or no EPP (median 3·6 [IQR 2·8–4·3] months between registration and randomisation). Thus, to make any comparison, 3·6 months would have to be added to the 14·4 months median survival in the EPP group of MARS and the resulting estimated 18 months survival from the start of treatment becomes similar to these reported series (table 3).

Panel **Research in context**

**Systematic review**

In 2004, we did a systematic review to assess the available evidence for effectiveness of extra-pleural pneumonectomy (EPP).^8^ We identified seven publications of multimodal therapy but all were analysed on the basis of completed treatment, offered no control data, and whether the most optimistic estimate of effect size was sufficient to outweigh the burden of treatment was debatable. During presentations of the available data to meetings at the British Thoracic Society and Society for Cardiothoracic Surgery in Great Britain and Ireland and in an editorial in the *British Medical Journal*^9^ we confirmed the uncertainty regarding the effectiveness of EPP was sufficient to propose a randomised controlled trial.

**Interpretation**

In the Mesothelioma and Radical Surgery (MARS) feasibility study, patients randomly assigned to no EPP had better median and 1-year survival than those assigned to EPP. When compared with survival data from EPP in a systematic review,^14^ the MARS surgical outcomes are in the middle of the reported range of median and 1-year survival rates. The no EPP group in MARS survived longer than the historical life expectancy estimates with which EPP results are reported in uncontrolled studies. In MARS, survival was reported from randomisation to EPP, which was after completion of three cycles of chemotherapy rather than from first chemotherapy as is customarily done for reports of EPP within trimodal therapy. An allowance for this difference in starting time puts the survival of the patients in the no EPP group in the upper part of the range of reported trimodal therapy outcomes. The evidence from MARS, in the context of external evidence from observational studies, suggests that the net effect of EPP is to shorten survival without a gain in quality of life. Lung-sparing surgery is associated with better outcomes than EPP^13^ but survival cannot be assumed to be better than it would have been without any extirpative surgery. A controlled trial of lung sparing surgery is needed.

**Table 3:** Reports of EPP within trimodal therapy

	**Start**	**End**	**Stage**	**Epithelioid histology (n/N)**	**Treatment**	**Number intended to treat**	**Median survival (intention to treat; months)**
Weder,^15^ 2007	2000	2003	T1–3, N0–2, M0	42/61	C, EPP, RT	61	19·8*
Rea,^16^ 2007	2000	2003	T1–3, N0–2, M0	20/21	C, EPP, RT	21	25·5*
Krug,^17^ 2009	2003	2006	T1–3, N0–2, M0	62/77	C, EPP, RT	77	16·8*
De Perrot,^18^ 2009	2001	2007	T1–3, N0–2, M0	44/60	C, EPP, RT	60§	14*
Van Schil,^19^ 2010	2005	2007	T1–3, N0–2, M0	31/58	C, EPP, RT	58	18·4†

EPP=extra-pleural pneumonectomy. TMT=trimodal therapy. C=chemotherapy. RT=radiotherapy.

* Counted from first chemotherapy.

† Counted from registration.

We cannot know from these non-controlled studies what survival might have been for similar patients to those having EPP but who were managed without surgery. The median survival for no EPP in MARS was 19·5 months from randomisation, which is after completion of chemotherapy. This is comparable with patients who received trimodal therapy (table 3), particularly if the discounted 3·6 months from registration to randomisation is taken into account. Overall survival for the no EPP group in MARS was better than that used in the power calculations for the proposed phase 3 trial, for which 670 patients would be needed on the basis of the power calculation.^9^ In any subsequent planned phase 3 study of extirpative surgery, this non-surgical outcome would have to be taken into consideration in the power calculations of the number of patients needed overall to show superiority in the surgical arm.

Morbidity is difficult to compare between arms with two very different approaches to management but for EPP morbidity has been consistently reported as high^19^, ^20^, ^21^ and MARS was no exception. In MARS, no significant differences were reported in the quality-of-life analyses between groups; however, there seemed to be poorer quality of life, particularly just after surgery in patients randomly assigned to EPP. This finding shows that any surgical group inevitably has more impaired quality of life in the first few weeks after surgery compared with a non-surgical group. The same consideration applies to any protocol that includes radical radiotherapy.

In MARS, most patients randomly assigned to EPP had surgery in two centres with considerable experience in the surgery and perioperative care of these patients. Late in the course of the study, two further surgical centres were approved to do EPP within MARS. No postoperative deaths occurred at these additional centres. The 30-day mortality rate was 10·5% (two of the 19 patients for whom surgery with intention to perform EPP was started). One further patient died from pneumonia 6 weeks postoperatively. In the systematic review of results of EPP in 34 studies,^14^ including 2320 patients, 30-day mortality ranged from 0% to 11·8% and was 6·0% overall. For the 993 patents in 14 studies that were reported since 2008 when MARS closed, and which therefore represent a similar era to when patients were recruited to MARS, mortality was 5·6% (range 0–11·1%).^14^ One should note, however, that for a hypothetical study of 20 consecutive operations, an anticipated 5% mortality would shift to 10% or 0% with one additional or one fewer death. Whether a death is recorded at 29 or 31 days would also make a disproportionately large difference to the results. In small series, such data are inherently unstable. In any future randomised phase 3 study of mesothelioma surgery, as many thoracic surgical centres as possible would need to be involved. In doing so, a rigorous method of surgical quality assurance would be important.

At the time MARS was being planned, pemetrexed was not yet the standard of care in the UK. The chemotherapy regimen for each patient was selected from the suggested regimens by the treating centre. During recruitment, the chemotherapy standard of care for mesothelioma changed, and patients recruited later were more likely to receive cisplatin and pemetrexed than those recruited earlier in the study. There was no imbalance in the use of pemetrexed between the EPP and no EPP arms.

The challenges of compliance with the trial protocol in this study should be taken into account when planning future phase 3 studies in which there is a large difference in treatments between the two arms. Despite having given informed consent to random allocation, some patients allocated to the no EPP group decided to pursue a radical surgery approach outside the trial. Some patients did not undergo surgery because, when reassessed after chemotherapy and when the risk:benefit balance of EPP had been explained by the operating surgeon, they opted not to proceed. Some patients had become inoperable during the time from initial assessment to reassessment. Nonetheless, although only 50 patients were randomised over the entire study period, rather than in the anticipated year, the fact that recruitment of these patients was possible suggests that the expected reluctance of patients to accept no radical surgery in a study with two very different treatment approaches to management of mesothelioma was not as marked as had been expected.

To our knowledge, MARS is the first study to successfully randomly assign patients to EPP and no radical surgery for mesothelioma. The MARS trial was rigorously done, with the final decision that a patient was eligible for randomisation within MARS made in discussions by the MARS virtual multidisciplinary team and the allocation to EPP or no EPP made within the ICR-CTSU. The accumulating outcome data were held at the trial centre and were only shared with the independent data monitoring committee. Although the study is small and the conclusions must be guarded, we believe the findings are of relevance to guide practice.

The median survival after EPP within MARS is consistent with 10, 12, 13, and 14 months in larger observational studies,^22^, ^23^, ^24^, ^25^ as was the proportion of complications. However, a much larger study with longer follow-up would be needed to provide reliable evidence on mortality patterns and long-term survival for any extirpative surgery for mesothelioma whether EPP or lung-sparing surgery. A trial assessing the potential benefits of total pleurectomy might be more practical in the future management of mesothelioma given the lower risk of perioperative mortality and morbidity in an ageing population with increasing comorbidities. On the basis of the results of MARS, a further study is being developed that does not include EPP as the recommended surgery. Recent data that compared lung-sparing total pleurectomy and decortication with EPP support the contention that this approach is unlikely to result in poorer survival than that associated with EPP in mesothelioma.^22^

## Web Extra Material

Supplementary webappendix
