# Supplementary material for: Extra-pleural pneumonectomy versus no extra-pleural pneumonectomy for patients with malignant pleural mesothelioma: clinical outcomes of the Mesothelioma and Radical Surgery (MARS) randomised feasibility study
Source: Lancet Oncol. 2011 Aug;12(8):763–72. doi: 10.1016/S1470-2045(11)70149-8 (PMC3148430; doi:10.1016/S1470-2045(11)70149-8)
Supplement: Supplementary webappendix [file mmc1.pdf]

THE LANCET Oncology

Supplementary webappendix

This webappendix formed part of the original submission and has been peer reviewed. We post it as supplied by the authors.

Supplement to: Treasure T, Lang-Lazdunski L, Waller D, et al for the MARS trialists. Extra-pleural pneumonectomy versus no extra-pleural pneumonectomy for patients with malignant pleural mesothelioma: clinical outcomes of the Mesothelioma and Radical Surgery (MARS) randomised feasibility study. *Lancet Oncol* 2011; published online July 1. DOI:10.1016/S1470-2045(11)70149-8

Web appendix for the MARS study

Surgical guidelines for the MARS study

Surgical procedure for patients allocated EPP surgery

Preoperative preparation
All patients should be ambulant and have adequate nutritional intake prior to surgery. Patients with significant pain secondary to chest wall involvement should be offered a pain service consultation and adequate pain control in order to achieve this.

Intra-operative management (recommended guideline checklist)

1. Aprotinin – Hammersmith protocol (½ dose) – started on all patients prior to skin incision, continued until ICU admission.

2. Broad spectrum antibiotic e.g. a cephalosporin – intra-operative antibiotic (with induction of anaesthesia) administered IV and continued for 5 days minimum.

3. Thoracic epidural catheter placed in all patients and maintained in situ for ~7 days unless patient is discharged earlier.

4. All patients have a large bore nasotracheal tube or oesophageal bougie 40 French placed to assist in oesophageal dissection.

5. Approach: A left or right thoracotomy as appropriate may be used with a single sixth intercostal space incision or a two level 4th and 8th space incision via a single skin incision. For right-sided disease a median sternotomy may be used in some cases.

6. If post-operative radiotherapy is planned, the true extent of the diaphragmatic resection and the anterior pleural reflection are marked with large clips at 1 to 1.5 cm intervals.

7. The diaphragm is reconstructed with a 2 mm Goretex™ soft tissue patch (or equivalent prosthetic material). Sutures are placed in the pericardium and diaphragm remnant, if possible. Some variations on suturing may apply from centre to centre.

8. The pericardium is reconstructed (both right and left) with Prolene™ mesh and Vicryl™ sutures (or appropriate substitute). Pericardial reconstruction on the left is necessary to avoid migration of the heart with significant contact to the left lateral chest wall (relevant to later planned radiation therapy).

9. A right angle 32 French tube thoracostomy is placed with a purse-string suture for closure of the insertion site at time of removal. This tube is placed to a balanced drainage post-pneumonectomy drainage device.

Post-operative discharge
Patients who do not live in the catchment area of the Surgical Centre may be transferred to their local centre post operatively 72 hours after notice of fitness to travel. Patients will be required to attend a follow-up out-patients appointment at the Surgical Centre approximately 6 weeks after their operation.

Post-operative radiotherapy
For patients allocated EPP surgery, post-operative radiotherapy should be directed at the empty hemithorax. If suitable radiotherapy equipment and expertise are not available at the Local Centre (LC), normal NHS practice should be followed whereby patients are referred to their nearest radiotherapy unit for this treatment. The surgical technique should be adjusted to facilitate postoperative hemi-thorax irradiation.

Management of patients allocated to no EPP surgery

1. Regular follow up by specialist.

2. Structured assessment of physical, psychological and social problems and appropriate treatment, which can include chemotherapy, palliative radiotherapy or other routine procedures.

3. Rapid referral to additional specialists when required.

4. Parallel nursing support.

Radiotherapy guidelines for the MARS study

These guidelines were based on the paper by Senan and van de Pol, 2004 [1]

Inclusion criteria
• Biopsy proven malignant mesothelioma
• Patients must have a pre-surgical performance score of WHO 0-1
• No prior history of chemotherapy or irradiation to the low neck, thorax or upper abdomen
• 18 years or older (providing the patient are otherwise fit to undergo the trimodality protocol)
• Normal renal function, and acceptable (predicted) post-radiotherapy renal function, as indicated by the creatinine clearance and isotope renography, with a relative contribution of the contralateral kidney of at least 40%
• Normal bilirubin and liver function tests (AST/ALT/Alkaline phosphatase < 1.5 N), and no history of liver cirrhosis. If the primary tumour is left sided, cirrhosis is not a contraindication
• Patients must be able to lie flat for the duration of the treatment planning sessions and treatment

• NOTE: Vaccination against Haemophilius influenza type B, Streptococcus pneumoniae and Neisseria meningitidis should be administered to all patients who will undergo a left–sided EPP as radiation-induced splenectomy is inevitable.

Inclusion criteria (post-surgery)
• Patients must have a performance score of WHO 0-2
• Patients will have recovered from surgery, and this will typically be 3-4 weeks post-surgery

• NOTE: All patients should be referred to a dietician and receive high calorie supplements prior to commencing radiotherapy.

Exclusion criteria
• extrathoracic metastases
• obvious invasion of mediastinal structures (heart, aorta, spine, esophagus, etc.)
• extensive chest wall invasion (resectable chest wall lesions are accepted)
• significant decrease in cardiac ejection fraction (< 40%) for patients with left-sided tumours
• contralateral or unresectable ipsilateral paratracheal lymph node involvement
• any positive intraperitoneal lesion
• positive intraperitoneal lavage

Protocol For Standard Radiotherapy

Target definition - CT scan
Patients will be positioned supine in a stable and reproducible position, e.g. on a lung board with arms above the head using an arm-support, for both CT scanning and daily treatments. Prior to the planning CT scan, all incision and drain sites will be marked using radio-opaque markers. (NOTE: Median steronotomy scars will NOT be included in the target volume.) The CT scan will extend from the cricoid cartilage to the pelvic brim, and the slice thickness will be 5mm at most. A treatment isocenter will preferably be tattooed at the time of CT scanning (i.e. not at a separate treatment simulation) in order to avoid simulator set-up errors.

Target volumes
Careful correlation of the pathology report with the operative findings, and a joint review of the contoured CTV with the thoracic surgeon, is strongly recommended. A standardized diagram to indicate sites of residual disease will drawn based upon input from the surgeon and pathologist, and will be made available to the radiation oncologist. The clinical target volume (CTV) includes the entire ipsilateral thoracic cavity from lung apex to insertion of the diaphragm, ipsilateral mediastinal pleura, mediastinal tissues at sites where evidence for tumour invasion was present, the ipsilateral pericardial surface, and full thickness of the thorax at the sites of thoracotomy and chest tube incisions. Chest wall bolus will be used at the sites of surgical incisions for at least a part of the treatment. A boost-CTV (bCTV) that encompasses sites of gross or microscopic residual disease should be separately contoured. A planning target volume (PTV) will be generated from the CTV and bCTV by the addition of a 3-dimensional margin of at least 1cm in order to account for internal mobility and set-up variations.

The planning target volume (PTV) will be generated from the CTV by the addition of a 3-dimensional margin of at least 1 cm in order to account for tumour motion, set-up variations and breathing.

Normal tissues
The spinal canal will be contoured and this will be taken to represent the cord.

Both kidneys, the oesophagus and entire liver will be contoured in all patients. If the treatment planning system does not allow for the import of large number of CT slices (e.g. for scans that extend to the pelvic brim in order to include the entire kidneys), a second planning CT scan of the abdomen should be performed. Treatment fields used for the hemithorax will be applied to the latter in order to derive dose-volume histograms of both kidneys and the liver.

The heart will be contoured on all slices; its cranial border will be the infundibulum of the right ventricle and the apex of both atria, and the great vessels will be excluded as much as possible. The caudal border is defined as the lowest part of the left ventricle's inferior wall that was distinguishable from the liver.

Normal tissue constraints for radiotherapy planning
i. The V20, which is the volume of healthy lung tissue receiving a total dose of ≥ 20 Gy [2] will be determined in all patients. The V20 values exceeding 15% will render a patient ineligible. All other factors remaining constant, the beam arrangement resulting in the lowest V20 will be chosen as the clinical plan.

ii. The dose to 80% of the contralateral (normally functioning) kidney should be less than 15 Gy.

iii. The mean dose to the liver should be below 35 Gy. When the mean hepatic dose is ≥ 35Gy, it is recommended that an underdosing of parts of the hemithorax without evidence of microscopic disease be accepted in order to
achieve this goal.

There is limited 3D data correlating radiation dose to healthy liver tissue with the risk of toxicity. No cases of radiation-induced liver disease were observed when the mean liver disease was <31 Gy, and it was consequently recommended that the dose to 50% of the liver should be 30 Gy or less.
However, these data were based on patients with irresectable intrahepatic malignancies, all of whom had concurrent intra-arterial chemotherapy, and 61 (of 203) patients had also received whole-liver radiotherapy.

iv. The dose to the spinal cord should not exceed 50 Gy (in fractions of 2 Gy).

v. Based on data derived in the era of 2-D radiotherapy planning, it has been recommended that 70% of the heart should receive less than 45 Gy, and that the maximum dose should not exceed 60 Gy. However, if residual disease is present in the cardiac region, it may be acceptable to deliver a full dose of 54 Gy to the heart

vi. No dose restrictions will be specified for the esophagus and dose-volume parameters will be recorded and correlated with observed toxicity.

Treatment planning
Only 3D radiotherapy planning (i.e. with beams-eye-view feature) will be used and treatment plans will be evaluated by review of dose-volume histograms.
If the cardiac or hepatic dose constraints cannot be met, a 2-phase treatment with a second plan using additional fields and a match-plane above the level of the organ at risk should be evaluated. One option would be to use wedged dorso-lateral photon fields together with an anterior electron field. Another option would be to incorporate a boost to only the bPTV, after a dose 45 Gy has been delivered to the entire hemithorax.

Fractionation scheme
PTV: A total of 54 Gy will be given in 30 once-daily fractions of 1.8 Gy (five fractions per week) to the planning target volume. An underdosing of a part of the PTV will be considered acceptable when the dose to critical organs exceeds the above mentioned tolerance doses. The dose in the PTV, however, should be at least 45Gy.

Boost PTV: The minimal accepted dose in the boost-PTV should be 54Gy.

Chest wall: A bolus will be used at chest wall incision sites for at least a part of the treatment in order to ensure that a dose of at least 45 Gy is delivered to this site.

Dose specification
The prescription dose will be specified at a relevant point in the PTV, and this is usually the iso-centre (intersection of the beams). The daily prescription dose will be 1.8 Gy using inhomogeneity corrections.

Dose recording
Dose volume histograms will be generated for the PTV, contralateral lung, kidneys, liver, spinal cord, heart and oesophagus. The following values should also be recorded:
- prescription point dose
- minimum, maximum and mean dose in PTV
- maximum dose to spinal cord
- V20

Dose homogeneity
The iso-dose curve representing the 95% of the prescription dose must encompass the entire planning target volume (ICRU 50 guidelines).

External beam equipment
Megavoltage equipment will be used with photon energies of 6-10 MV. A multileaf collimator or standard blocks will be used to shape the irradiation portal according to the target volume.

Treatment verification
Portal imaging films or electronic portal images must be obtained during the course of treatment in accordance with the standard department protocols. Where no protocol exists, portal imaging should be performed weekly and compared with digitally reconstructed images from the planning CT scan. In the absence of a setup correction protocol, any discrepancies exceeding 5 mm will be corrected for.

Radiation toxicity and scoring
Radiation toxicity will be scored according to the CTC criteria [3]

Potential risks
Likely acute toxicities include: oesophagitis, nausea/vomiting, weight loss, fatigue, neutropenia/anemia and cough. Potential long-term toxicities include oesophageal dysmotility/stricture, pericarditis, cardiomyopathy, pneumonitis/dyspnea on exertion/shortness of breath at rest and damage to the liver or kidneys.

References

1. Senan S, van de Pol M. Considerations for post-operative radiotherapy to the hemithorax following extrapleural pneumonectomy in malignant pleural mesothelioma. *Lung Cancer* 2004; 45 Suppl 1: S93-S96

2. Senan S, De Ruysscher D, Giraud P, Mirimanoff R, Budach V; Radiotherapy Group of European Organization for Research and Treatment of Cancer. Literature-based recommendations for treatment planning and execution in high-dose radiotherapy for lung cancer. *Radiother Oncol.* 2004; 71(2): 139-46

3. Cancer Therapy Evaluation Program, Common Terminology Criteria for Adverse Events, Version 3.0, DCTD, NCI, NIH, DHHS March 31, 2003 (http://ctep.cancer.gov) . Publish Date: June 10, 2003.

MARS Study Investigators and Centre Details

Principal and main co-investigators according to centre (number of patients registered) were:

Guy's and St. Thomas' Hospital, London (40) D Landau, L Lang-Lazdunski; Royal Marsden NHS Foundation Trust, Sutton and London (15) M O'Brien; Weston Park Hospital, Sheffield (14) M Hatton; Papworth Hospital, Cambridge (9) R Rintoul; Glenfield Hospital, Leicester (8) G Thomas, D Waller J Entwisle; St. James's Hospital / Cookridge, Leeds (7) M Snee, K Papagiannopoulos; Birmingham Heartlands (7) J Thompson; Royal Berkshire Hosptial, Reading (4) R Brown; Bristol Royal Infirmary (3) A Morgan*; Nottingham City Hospital (3) S Morgan; Queens Hospital, Burton on Trent (2) P Beckett.
*Deceased

Principal investigators at additional centres conducting specialist radiotherapy:
Addenbrooke's Hospital Cambridge, K Waite; Queen Elizabeth Birmingham, A Chetiyawardana.

Surgeons at additional centres conducting specialist EPP surgery:
Royal Brompton, London E Lim, M Dusmet; Northern General Hospital, Sheffield J Edwards.
